# Supplementary material for: Physicochemical and Stability Evaluation of Topical Niosomal Encapsulating Fosinopril/γ-Cyclodextrin Complex for Ocular Delivery
Source: Pharmaceutics. 2022 May 27;14(6):1147. doi: 10.3390/pharmaceutics14061147 (PMC9228017; doi:10.3390/pharmaceutics14061147)
Supplement: Supplementary file 1 [file pharmaceutics-14-01147-s001.zip › pharmaceutics-1723216-supplementary.pdf]

pharmaceutics

Supplementary Materials: Physicochemical and Stability Evaluation of Topical Niosomal Encapsulating Fosinopril/γ-Cyclodextrin Complex for Ocular Delivery

Hay Marn Hnin, Einar Stefánsson, Thorsteinn Loftsson, Rathapon Asasutjarit, Dusadee Charnvanich and Phatsawee Jansook

Table S1. pH and zeta potential values of FOS niosomal preparation and FOS/γCD complex storage at 4 °C, 30 ± 2 °C (75 ± 5% RH) and 40 ± 2 °C (75 ± 5% RH) for 0, 1,3 and 6 months (n = 3, mean ± SD).

Time (month)	Formulation					
	pH			zeta potential (mV)		
	Sp-DCP	Sp-DCP+γCD	FOS/γCD complex	Sp-DCP	Sp-DCP+γCD	FOS/γCD complex
4 °C						
Initial	6.82 ± 0.02	6.72 ± 0.02	7.41 ± 0.01	−35.15 ± 1.48	−25.83±0.87	−11.13±0.62
1 Month	6.76 ± 0.02	6.68 ± 0.01	7.32 ± 0.02	−35.14 ± 1.89	−25.35±0.06	−10.87±0.46
3 Months	6.71 ± 0.04	6.63 ± 0.02	7.10 ±0.02	−35.09 ± 0.09	−25.22±1.65	−10.74±0.47
6 Months	6.63 ± 0.02	6.52 ± 0.04	6.78 ±0.05	−34.92 ± 2.15	−25.12±1.65	−10.45±0.47
30 ± 2 °C (75 ± 5% RH)						
Initial	6.82 ± 0.02	6.72 ± 0.02	7.41 ± 0.01	−35.15±1.48	−25.83 ± 0.87	−11.13 ± 0.62
1 Month	6.57 ± 0.03	6.53 ± 0.03	5.60 ± 0.02	−35.10±1.75	−25.23 ± 0.89	−10.97 ± 0.66
3 Months	6.32 ± 0.11	5.53 ± 0.03	5.34 ± 0.06	−35.07±1.11	−25.17 ± 1.34	−10.73 ± 0.14
6 Months	4.39 ± 0.03	4.58 ± 0.03	4.70 ± 0.01	−32.63±1.19	−21.53 ± 0.88	−9.53 ± 0.14
40 ± 2 °C (75 ± 5% RH)						
Initial	6.82 ± 0.02	6.72 ± 0.02	7.41 ± 0.01	−35.15 ± 1.48	−25.83 ± 0.87	−11.13 ± 0.62
1 Month	5.88 ± 0.04	5.56 ± 0.03	5.11 ± 0.04	−27.77 ± 0.85	−23.80 ± 1.56	−9.50 ± 0.56
3 Months	4.42 ± 0.09	4.64 ± 0.01	4.89 ± 0.01	−25.97 ± 1.30	−22.45 ± 0.52	−8.35 ± 0.50
6 Months	4.23 ± 0.10	4.39 ± 0.04	4.49 ± 0.04	−23.87 ± 1.89	−19.95 ± 0.71	−8.02 ± 0.86

Table S2. Average particle size and size distribution (PDI) of FOS niosomal preparation and FOS/γCD complex storage at 4 °C, 30 ± 2 °C (75 ± 5% RH) and 40 ± 2 °C (75 ± 5% RH) for 0, 1,3 and 6 months (n = 3, mean ± SD).

Time (month)	Formulation					
	Average particle size (nm)			Size distribution (PDI)		
	Sp-DCP	Sp-DCP+γCD	FOS/γCD complex	Sp-DCP	Sp-DCP+γCD	FOS/γCD complex
4°C						
Initial	275.10 ± 48.20	241.70 ± 34.40	243.60 ± 45.60	0.44 ± 0.02	0.18 ± 0.01	0.51 ± 0.01
1 Month	281.20 ± 7.40	258.03 ± 8.80	282.00 ± 26.20	0.44 ± 0.01	0.24 ± 0.03	0.51 ± 0.07
3 Months	306.80 ± 8.85	266.45 ± 22.86	768.35 ± 134.22	0.55 ± 0.07	0.42 ± 0.05	0.53 ± 0.06
6 Months	435.67 ± 19.48	421.47 ± 10.34	2330.75 ± 296.58	0.64 ± 0.03	0.59 ± 0.02	0.55 ± 0.01
30 ± 2−°C (75 ± 5% RH)						
Initial	275.10±48.20	241.70 ± 34.40	243.60 ± 45.60	0.44 ± 0.02	0.18 ± 0.01	0.51 ± 0.01
1 Month	293.30±27.10	254.70 ± 10.10	1019.40 ± 179.3	0.45 ± 0.10	0.29 ± 0.10	0.59 ± 0.01
3 Months	391.40±7.69	309.57 ± 13.63	1023.20 ± 65.15	0.52 ± 0.02	0.53 ± 0.07	0.75 ± 0.05
6 Months	911.85±43.73	452.27 ± 26.40	3244.75 ± 216.10	0.71 ± 0.08	0.74 ± 0.10	0.78 ± 0.16
40 ± 2 °C (75 ± 5% RH)						
Initial	275.10 ± 48.20	241.70 ± 34.40	243.60 ± 45.60	0.44 ± 0.02	0.18 ± 0.01	0.51 ± 0.01
1 Month	296.20 ± 7.40	277.80 ± 10.40	1607.20 ± 285.10	0.48 ± 0.10	0.33 ± 0.10	0.79 ± 0.10
3 Months	380.80 ± 14.07	360.10 ± 47.86	1846.70 ± 91.79	0.73 ± 0.01	0.53 ± 0.09	0.88 ± 0.08
6 Months	1895.00 ± 511.53	1071.10 ± 140.27	3942.70 ± 420.90	1.00 ± 0.00	0.95 ± 0.05	0.97 ± 0.24
